# Novel CRISPR/Cas9 system assisted by fluorescence marker and pollen killer for high‐efficiency isolation of transgene‐free edited plants in rice

**DOI:** 10.1111/pbi.14293

**Published:** 2024-02-01

**Authors:** Dong Yu, Tianshun Zhou, Na Xu, Xuewu Sun, Shufeng Song, Hai Liu, Zhizhong Sun, Qiming Lv, Jin Chen, Yanning Tan, Xiabing Sheng, Li Li, Dingyang Yuan

**Affiliations:** ^1^ State Key Laboratory of Hybrid Rice Hunan Hybrid Rice Research Center, Hunan Academy of Agricultural Sciences Changsha China; ^2^ Longping Branch, College of Biology Hunan University Changsha China

**Keywords:** CRISPR/ Cas9, Transgene‐free, Fluorescence Marker, Pollen Killer, Rice

CRISPR/Cas9 gene‐editing technology has revolutionized functional genomics and crop genetic improvement due to its simplicity, efficiency and stability (Gao, 2021). Nevertheless, the presence of CRISPR/Cas9 elements in the edited offspring increases the likelihood of inducing unstable and unpredictable phenotypes, posing challenges for commercial and research applications owing to the ongoing editing activity of the Cas9 protein (He *et al*., 2018). Typically, transgene‐free mutants are isolated from edited progeny using time‐consuming and labour‐intensive methods such as backcrossing and PCR amplification. Recently, several CRISPR/Cas9 assistant selection systems have emerged that integrate CRISPR/Cas9 with reporter genes to visually identify transgene‐free edited offspring (Liu *et al*., 2019; Yan *et al*., 2021). Notably, we can only separate 25% of the transgene‐free progeny (He *et al*., 2018) from a single‐copy hemizygote plant, whereas we cannot separate transgene‐free plants from homozygote plants. Alternatively, transgene‐killer CRISPR systems (TKC), assisted by using the *CMS2* gene and *BARNASE* (He *et al*., 2018; Liu *et al*., 2022), which eliminate the male and female gametes harbouring them, having been developed to obtain transgene‐free T_1_ plants. However, the complete lethality of transgenic gametes prevents the transmission of the TKC element to the progeny genome and hinders multi‐target fully edited mutant generation (Liu *et al*., 2022). Therefore, achieving a balance between the separation efficiency of transgene‐free seeds and obtaining a portion of transgenic seeds is crucial for sustaining the ongoing editing effect of Cas9 and facilitating cross‐generational editing breeding (Wang *et al*., 2023).

In this study, we developed a novel CRISPR/Cas9 system called the fluorescence marker and pollen killer‐assisted CRISPR/Cas9 system (FMPKC) for cross‐generational editing and efficient isolation of transgenic‐free rice plants (Figure 1a). The FMPKC system employed a *DsRed2* fluorescence marker to distinguish transgenic offspring, in linkage with a pollen killer (*RAmy1A* or *orfH79*) to increase the proportion of non‐transgenic seeds (Figure 1a–e). Additionally, the system integrates the Golden Gate cloning method with an innovative Cyclic Digestion and Assembly method (Figure S1). These methods improved the flexibility and efficiency of gRNA expression cassettes (gRECs) assembly (Figure S2). We used the FMPKC system to edit the internode length gene *EUI1* and pollen fertility gene *PTC1* in rice. For the FMPKC1‐EUI1 construct, four gRECs were assembled using Cyclic Digestion and Assembly, whereas two gRECs were ligated into the FMPKC2‐PTC1 construct using Golden Gate cloning. Following *A. tumefaciens*‐mediated transformation of cultivars ZH11 and YG13, five FMPKC1‐EUI1 and nine FMPKC2‐PTC1 positive lines were obtained (Figure S3). We analysed the phenotypes of the 14 positive lines. The measurement of internode length and plant height demonstrated significant elongation in four of the FMPKC1‐EUI1 positive lines, whereas one line (eui1 #4) was unchanged compared to the wild‐type plants. Among the nine FMPKC2‐PTC1 positive lines, three were male sterile and six were normal (Table S1). Screening for seed fluorescence from the 14 T_0_‐positive plants, demonstrated that the proportion of non‐fluorescent seeds in all lines, except eui1 #1, was approximately 50% (Figure S4a). This indicates that the inserted T‐DNAs were present in a single‐copy hemizygous state. In agreement with this, Droplet Digital PCR analyses demonstrated that, except for eui1 #1, the number of exogenous elements in all 13 transgenic lines was half of that in the single‐copy homozygous reference gene *OsSPS1* (Figure S4c; Table S2). This also strongly suggests that T‐DNA was mainly inserted into the genome of T_0_ transgenic‐positive plants in a single‐copy hemizygous state.

**Figure 1:**
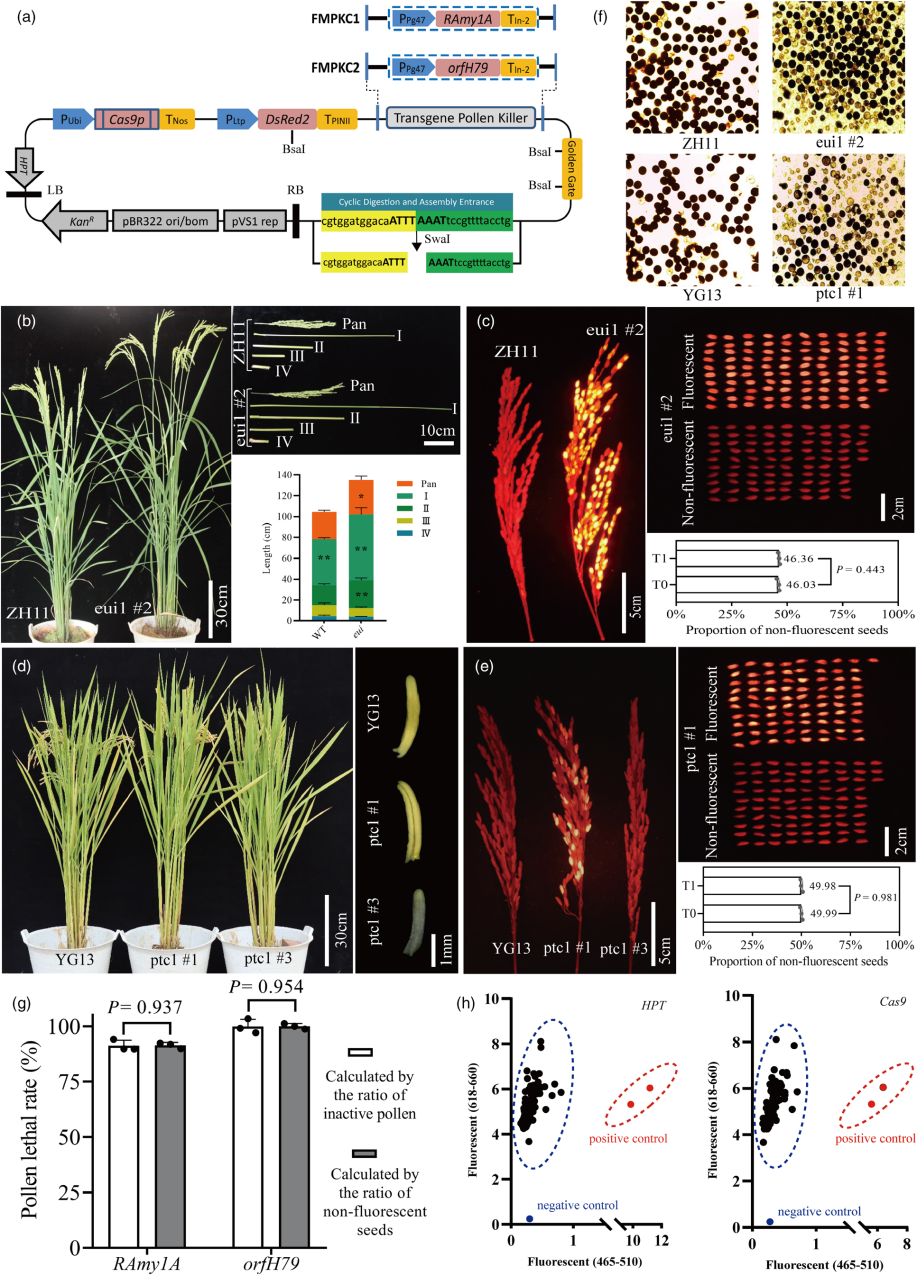
FMPKC system designed to target edited rice internode length gene *EUI1* and pollen fertility gene *PTC1*. (a) Schematic representation of the FMPKC plasmid. (b) Plant height and internode length phenotypes of wild‐type ZH11 and T_0_ eui1 #2 plants. (c) Proportion of non‐fluorescent seeds of *eui1* #2 edited by FMPKC1‐EUI1. (d) Plant type and anther morphology of the wild‐type YG13 and T_0_ generation lines ptc1 #1 (fertile) and ptc1 #3 (male sterile without seeds), respectively. (e) Proportion of non‐fluorescent seeds of ptc1 #1 edited with FMPKC2‐PTC1. (f) Pollen activities of the T_1_ lines eui1 #2 and ptc1 #1 were detected using I‐KI staining. (g) Pollen lethality efficiency of *RAmy1A* and *orfH79*. (h) Detection of *HPT* and *Cas9* genes in plant populations developed from non‐fluorescent seeds using the KASP genotyping technology.

Statistical analysis revealed that the proportion of non‐fluorescent seeds in the FMPKC‐EUI1 plants, excluding eui1 #1, was slightly lower than that in the FMPKC‐PTC1 plants (Figure 1c,e). This variation in non‐fluorescent seed frequency could be attributed to a difference in the mortality rates of the pollen killer *RAmy1A* in FMPKC1‐EUI1 and *orfH79* in FMPKC2‐PTC1. To calculate the lethality rates of the two pollen killers, we examined the pollen activity of FMPKC1‐EUI1 hemizygotes and FMPKC2‐PTC1 fertile hemizygotes. The proportion of active pollens in FMPKC1‐EUI1 edited plants was slightly higher than that in FMPKC2‐PTC1 edited plants (Figure 1f). The *RAmy1A* lethality rate was 91.22%, whereas that of *orfH79* was 99.85% as estimated from the inactive pollen ratio of the T_1_ generation. The pollen lethality rates were 91.35% for *RAmy1A* and 99.98% for *orfH79* when calculated using the fluorescent seed ratio of the T_1_ generation, which were highly consistent with those obtained using the inactive pollen ratio method (Figure 1g). Furthermore, there were no statistically significant differences in the lethality rates of *RAmy1A* and *orfH79* between the T_1_ and T_2_ generations (Figure S5). These findings indicate that *orfH79* can eliminate transgenic pollen, whereas *RAmy1A* has lower pollen inactivation efficiency. Notably, compared to single reporting systems, FMPKC increased the proportion of non‐fluorescent seeds in the edited offspring from 25% to approximately 50%, assisted by either pollen killer. Moreover, no exogenous transgenic elements were detected using the KASP technique in T_1_ generation plants developed from non‐fluorescent seeds (Figure 1h), illustrating that our FMPKC system can significantly enhance transgenic‐free plant production.

Further investigation revealed that ptc1 #1 and eui1# 4, which lacked phenotypic variation in the T_0_ generation, exhibited abnormal increases in the number of offspring with mutant traits. Notably, the proportion of T_1_ plants with mutant traits that developed from fluorescent seeds was significantly higher than that derived from non‐fluorescent seeds (Table S1). This implies that fluorescent seeds inherit hemizygous CRISPR/Cas9 elements, enabling continuous editing of the target sites during fluorescent seed development and resulting in an increase in mutated plants. To confirm this hypothesis, Sanger sequencing was performed to analyse the mutation types in the different generations of ptc1 #1 and eui1 #4. Heterozygous mutations in PTC1‐Target1 and EUI1‐Target3 were exclusively observed in ptc1 #1 and eui1 #4, respectively (Table S3). Consequently, they did not exhibit any mutant phenotypes in the T_0_ generation. However, other targets exhibited mutations in varying proportions in the T_1_ generation. Finally, we sequenced and obtained homozygous double and quadruple mutants from offsprings in the T_1_ generation of ptc1 #1 and the T_2_ generation of eui1 #4, respectively (Figure S6). The T‐DNA of FMPKC was inherited in a single‐copy hemizygous state, thereby preserving the continuous editing effect of Cas9 on target sites during the fluorescent progeny development and facilitating cross‐generation editing to obtain plants with all targets edited (Figure S7).

In conclusion, we developed an FMPKC system to efficiently screen non‐transgenic edited plants and demonstrated that the FMPKC system is highly suitable for multi‐target or multi‐gene editing because of its cross‐generation editing capability. Our two examples demonstrate that it has great potential for application in rice functional genomics and breeding improvement in the future.

## Funding

This work was supported by the Special Funds for Construction of Innovative Provinces in Hunan Province (2021NK1002), Natural Science Foundation of Hunan Province (2023JJ30444), Major Science and Technology Program in Hainan Province (ZDKJ2021002) and Postgraduate Scientific Research Innovative Project of Hunan Province (QL20220107).

## Supporting information


**Data S1** Materials and methods.
**Figure S1-S8** Supplementary Figures.
**Table S1-S4** Supplementary Tables.

## Data Availability

The data that support the findings of this study are openly available in PBI‐00905‐2023.R1 at https://mc.manuscriptcentral.com/plantbiotechjournal?URL_MASK=9246aa6b6c8c4bcda55b1826deeddc30, reference number PBI‐00905‐2023.R1.
