# Supplementary material for: Novel CRISPR/Cas9 system assisted by fluorescence marker and pollen killer for high‐efficiency isolation of transgene‐free edited plants in rice
Source: Plant Biotechnol J. 2024 Feb 1;22(6):1649–51. doi: 10.1111/pbi.14293 (PMC11123406; doi:10.1111/pbi.14293)
Supplement: Supplementary file 1 — Data S1 Materials and methods. Figure S1-S8 Supplementary Figures. Table S1-S4 Supplementary Tables. [file PBI-22-1649-s001.docx]

**Supporting Information**

**Novel CRISPR/Cas9 System Assisted by Fluorescence Marker and Pollen Killer for High-Efficiency Isolation of Transgene-Free Edited Plants in Rice**

Dong Yu^1†^, Tianshun Zhou^1,2†^, Na Xu^1†^, Xuewu Sun^1^, Shufeng Song^1^, Hai Liu^1^, Zhizhong Sun^1^, Qiming Lv^1^, Jin Chen^1^, Yanning Tan^1^, Xiabing Sheng^1^, Li Li^1,2*^ and Dingyang Yuan^1,2*^

^1^ State Key Laboratory of Hybrid Rice, Hunan Hybrid Rice Research Center, Hunan Academy of Agricultural Sciences, Changsha, 410125, China

^2^ Longping Branch, College of Biology, Hunan University, Changsha, 410125, China.

* Correspondence: [yuandingyang@hhrrc.ac.cn](mailto:yuandingyang@hhrrc.ac.cn) (Dingyang Yuan); [lili@hhrrc.ac.cn](mailto:lili@hhrrc.ac.cn) (Li Li)

† These authors are co-first authors

**Supplemental Figures**


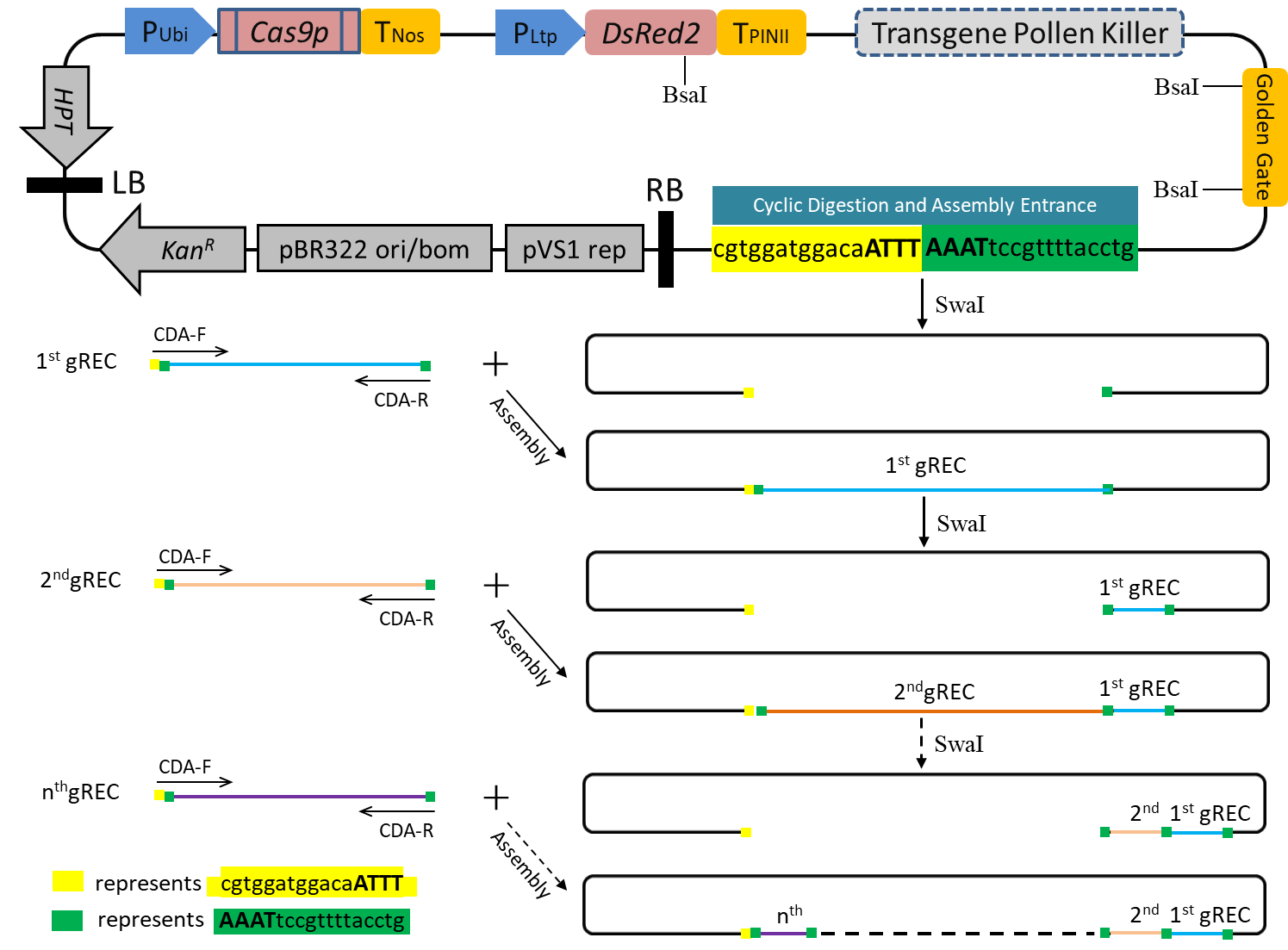


**Figure S1 Schematic diagram of Cyclic Digestion and Assembly**

The primer CDA-F for gRNA expression cassettes (gREC) amplification contains a Cyclic Digestion and Assembly entrance sequence, whereas CDA-R has only half an entrance sequence; therefore, an identical assembly entrance will be regenerated for the next gREC assembly after the previous gREC is assembled into the FMPKC vector (the fluorescence marker and pollen killer-assisted CRISPR/Cas9 vector). Thus, multiple gRECs can be gradually inserted into FMPKC by the cyclic operation of SwaI digestion and Gibson Assembly. After testing, up to seven gRECs were assembled into the FMPKC vectors in a stepwise manner (**n ≤7**).


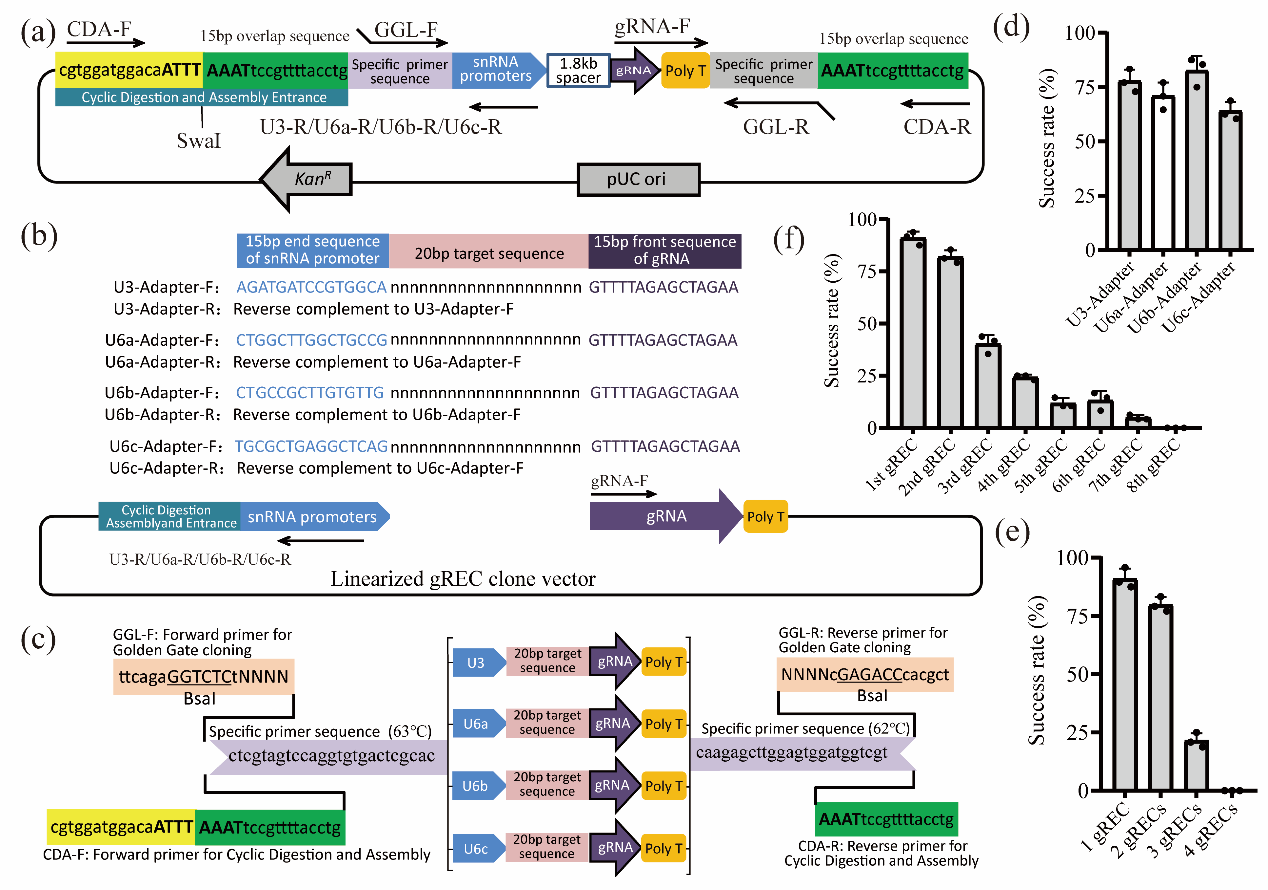


**Figure S2 Construction method and assembly efficiency of gRNA expression cassettes (gRECs) cloning vector**

(a) Schematic representation of gRECs vector clones. The gRNA was driven by the rice small nuclear RNA (snRNA) promoters U3, U6a, U6b, and U6C to form four gRECs cloning vectors: pCR-U3, pCR-U6a, pCR-U6b, and pCR-U6C. The primers gRNA-F and U3-R (U6a-R, U6b-R, or U6c-R) were designed at the end of the snRNA promoter and in front of the gRNA scaffold to amplify the linearized gREC cloning vector. A 1.8-kb interval sequence was inserted between the snRNA promoter and gRNA, which was used to prevent the amplification products from forming circular plasmids by controlling the extension time. The target site sequences were synthesized into 50-bp double-stranded DNA adapters and then assembled into linearized cloning vectors using Gibson Assembly to form a complete gRNA expression cassette. The specific primer sequence, Cyclic Digestion and Assembly entrance, and a 15-bp overlapping sequence were introduced into both ends of the gRNA expression cassettes, which was convenient for designing GCL-F/GCL-R and CDA-F/CDA-R primers for amplifying complete gRECs with targets. Complete gRECs amplified using primers GGL-F/GGL-R were ligated to FMPKC by Golden Gate cloning, and the CDA-F/CDA-R-amplified gRECs were assembled into FMPKC by Cyclic Digestion and Assembly. (b) Sequence structure of the target site adapter. The target site adapter was a synthetic double-stranded DNA comprising a 15-bp snRNA promoter end sequence, a 20-bp target sequence, and a 15-bp gRNA front sequence. (c) Complete gRNA expression cassette sequence and amplified primer sequence structure. (d) Success rates of assembling target site adapter into four gREC cloning vectors. (e, f) Success rates of inserting gRECs into the FMPKC vector using Golden Gate cloning and Cyclic Digestion and Assembly, respectively. Data are shown as mean ± standard deviation.


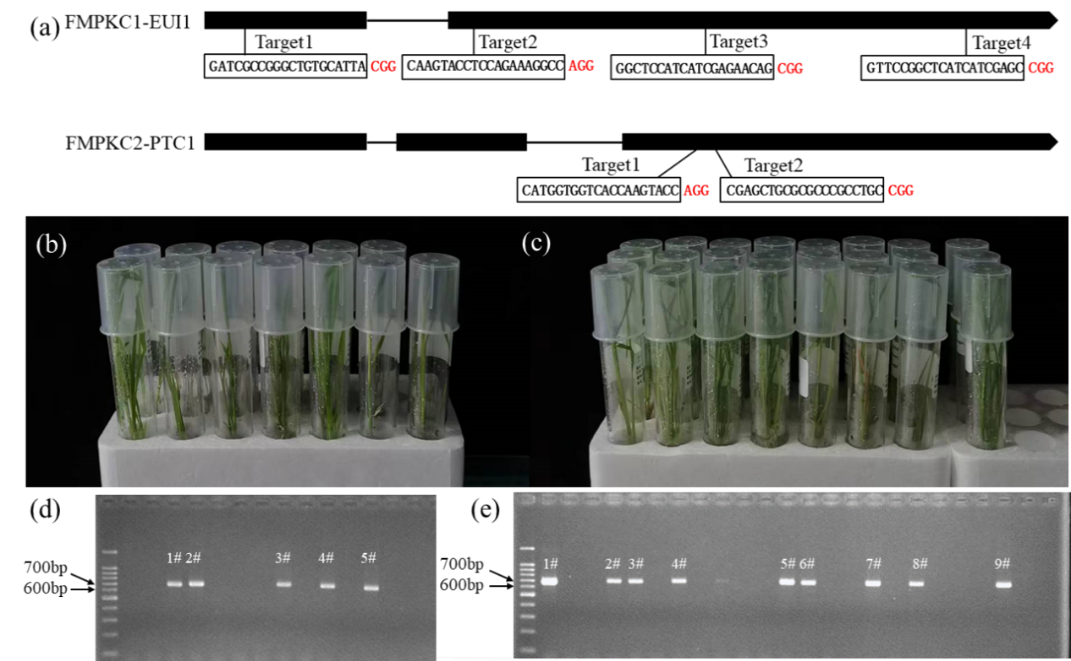


**Figure S3 Design of targets and detection of positive transgenic plants**

(a) Targets of FMPKC1-EUI1 and FMPKC2-PTC1. Bases in red indicate PAM sequences. (b, c) The T_0_ transgenic lines of 13 FMPKC1-EUI1 and 24 FMPKC2-PTC1 were obtained by the *A. tumefaciens*-mediated genetic transformation method. (d, e) *DsRed2* gene detection of FMPCK1-EUI1 and FMPCK2-PTC1 T_0_ positive plants, respectively.


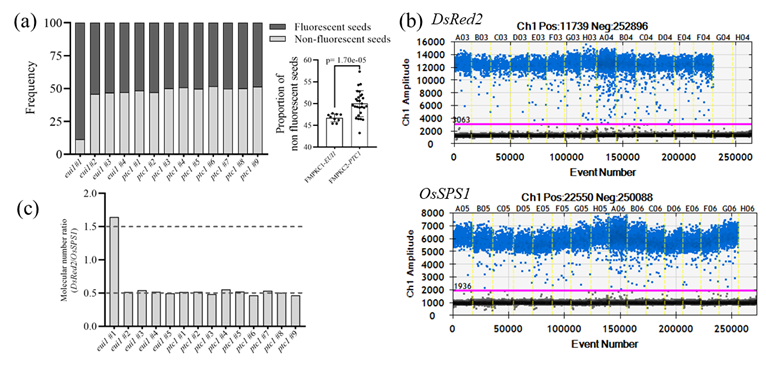


**Figure S4 Copy numbers of FMPKC T-DNA region in T**_0_ **transgenic positive lines**

(a) Proportions of fluorescent seeds to non-fluorescent seeds in T_0_ positive transgenic plants. eui #1 was not included in the calculation of the average proportion of non-fluorescent seeds in FMPKC-EUI1. Three panicles were taken from each plant to analyze the average proportion of non-fluorescent seeds and data are shown as mean ± standard deviation. Significant differences based on the Student’s t-test. (b) Detection of *DsRed2* and *OsSPS1* copy numbers by droplet digital PCR (ddPCR). *OsSPS1* is a single-copy gene in the rice genome and was used as control. (c) Molecular number ratios of *DsRed2* and *OsSPS1*, which is indicative of FMPKC T-DNA copy number per genome.


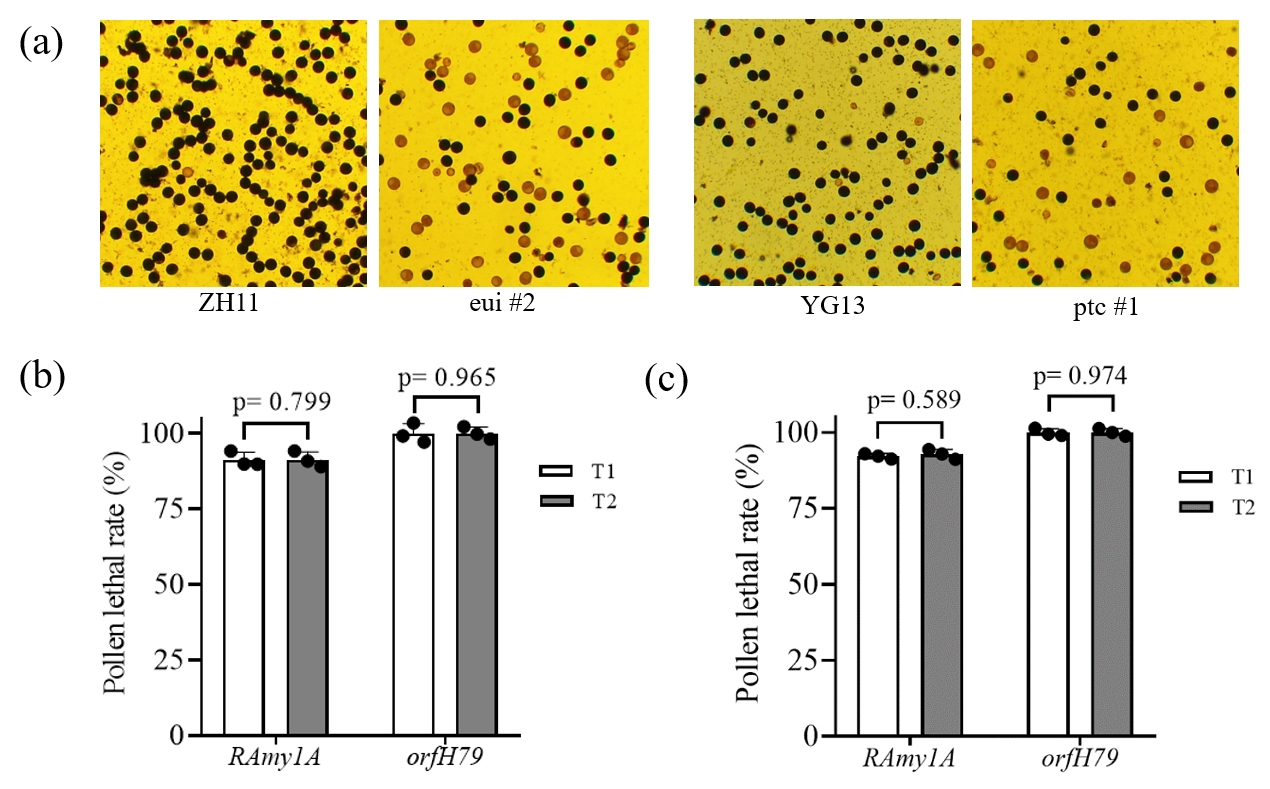


**Figure S5 Lethality rates of *RAmy1A* and *orfH79* did not exhibit significant differences across different generations**

(a) Pollen activities of T_2_ line eui1 #2 and ptc1 #1 were detected by I-KI staining. (b) Lethality rates of *RAmy1A* and *orfH79* did not differ significantly between the T_1_ and T_2_ generations when assessed based on the inactive pollen proportion. (c) Lethality rates of *RAmy1A* and *orfH79* did not differ significantly between the T_1_ and T_2_ generations when assessed based on the non-fluorescent seed proportion.


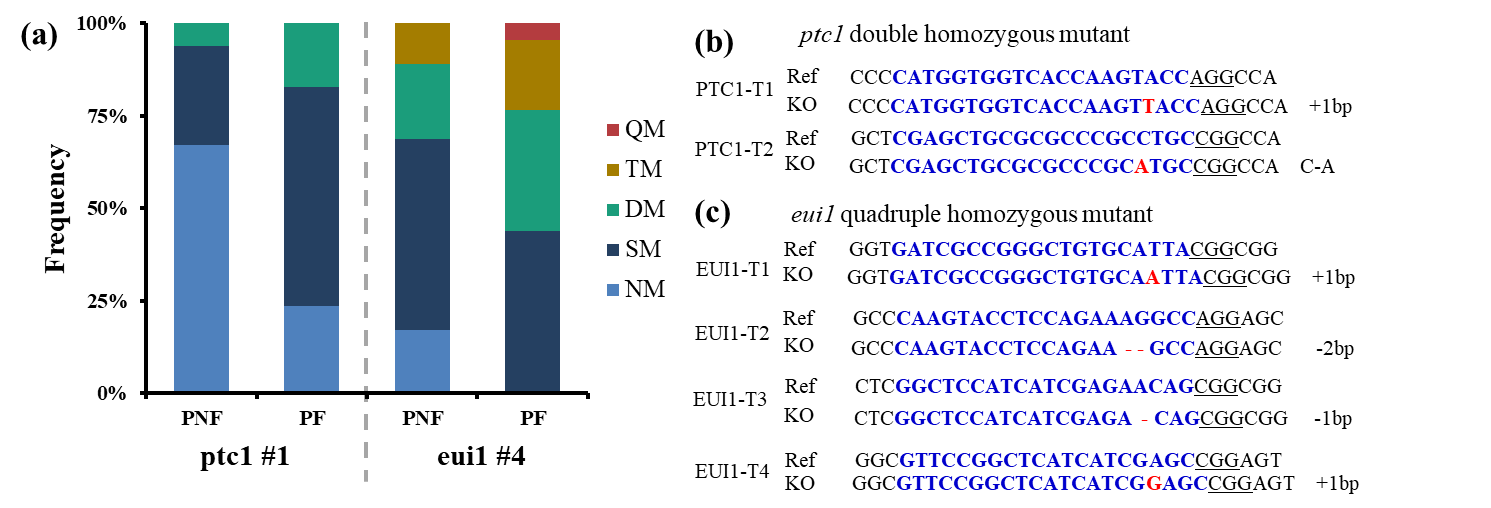


**Figure S6 Highly efficient creation of multi-target mutants with FMPKC system**

(a) Mutation site analysis of T_1_ plants grown from seed of ptc1 #1 and eui1 #4. The targets were considered to be edited once target sequences were changed without considering the mutation type. (b) Double homozygous transgenic-free mutant screen from T_1_ generation of ptc1 #1. (c) Quadruple homozygous transgenic-free mutant screen from T_2_ generation of eui1 #4. Nucleobases with an underline are PAM sequences. Nucleobases in blue and red indicate target sequences and mutated sequences, respectively. PNF, Plants grown from non-fluorescent seeds; PF, Plants grown from fluorescent seeds; NM, no mutation; SM, single mutation; DM, double mutation; TM, triple mutation; QM, quadruple mutation.


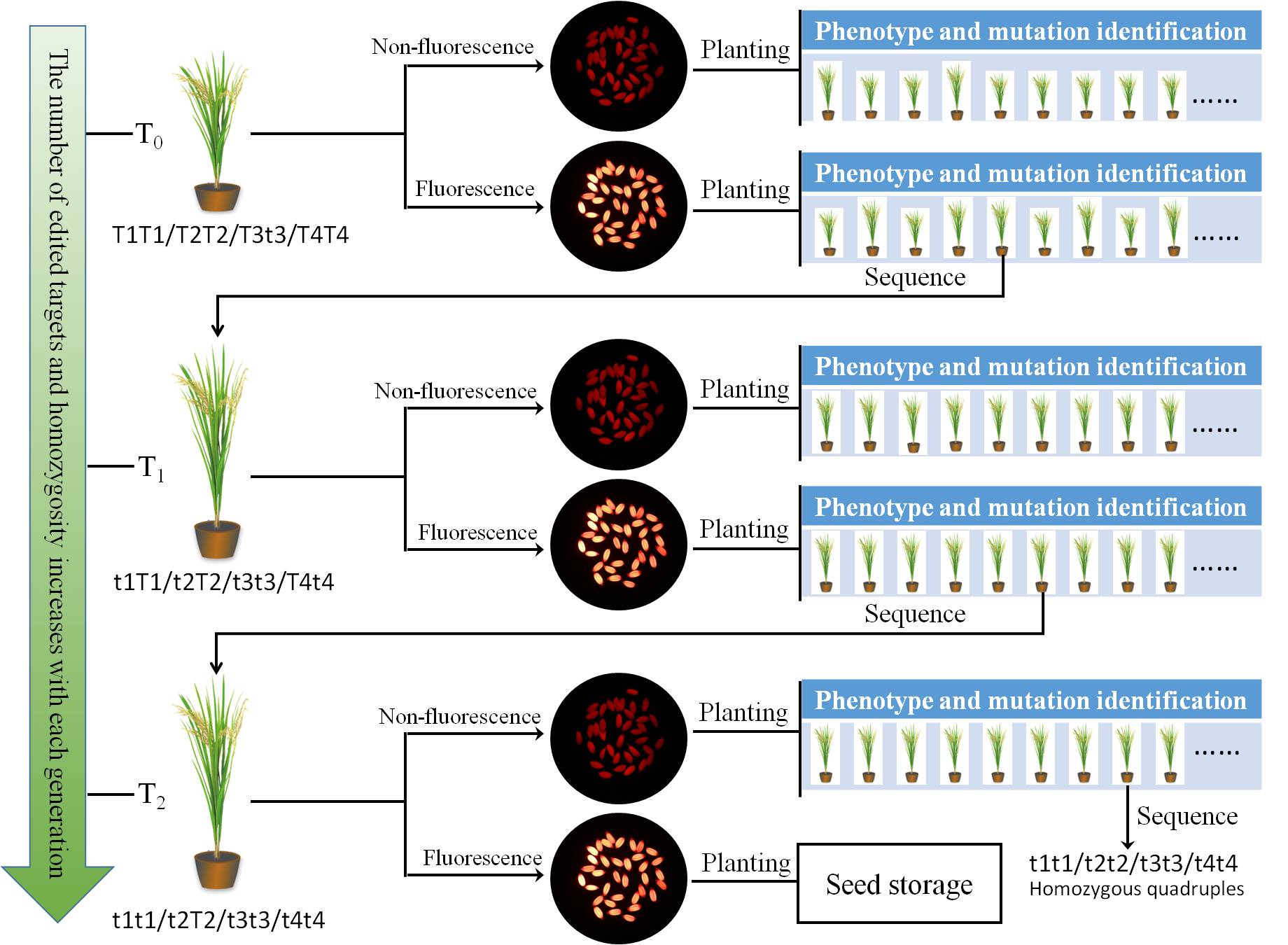


**Figure S7 Cross-generation editing to obtain multiple targets of all edited plants by the FMPKC system**

Fluorescent seeds (FMPKC hemizygotes) were continuously edited using the Cas9 element, and the number and homozygosity of edited targets in the progeny increased with successive generations. Finally, multi-target-edited homozygous mutants without transgenes, such as homozygous quadruples (t1t1/t2t2/t3t3/t4t4t), were obtained from the offspring of non-fluorescent seeds. Uppercase "T" means that the target sequence had no editing mutation, and lowercase "t" means that the target sequence had an editing mutation. P^n^ = 1/ (4-2R), where P^n^ is the proportion of non-fluorescent seeds, and R is the lethality rate of pollen killers. T_0_, T_0_ generation; T_1_, T_1_ generation; T_2_, T_2_ generation. T1, Target1; T2, Target2; T3, Target3; T4, Target4.


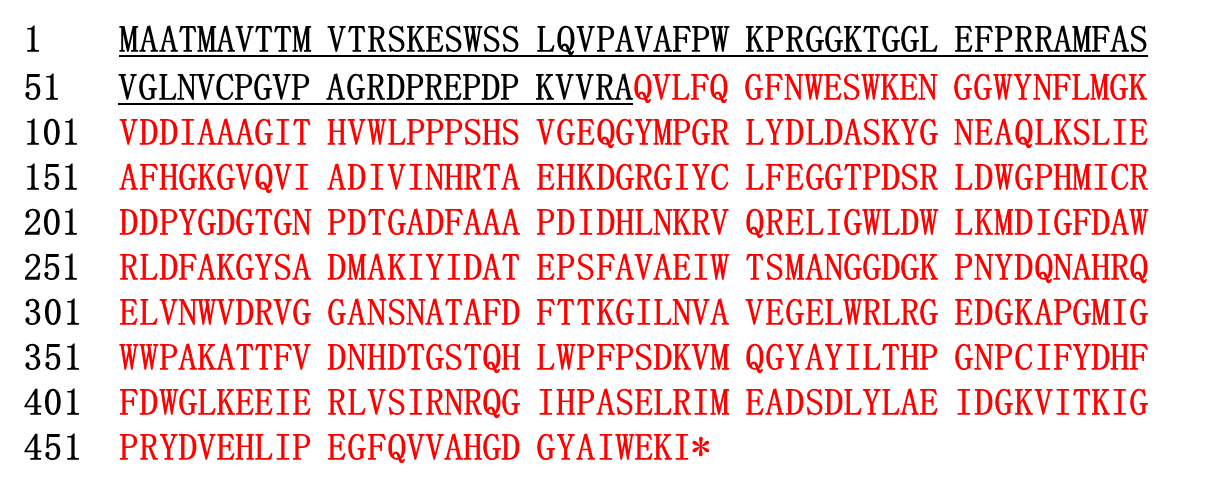


**Figure S8 Amino acid sequence of the modified RAmy1A Protein**

The underlined region represents the maize Brittle-1 transit peptide (75 aa). The red region represents the RAmy1A protein sequence that lacks a signal peptide.

**Supplemental Tables**

**Table S1 Phenotypic investigation of T_0_ and T_1_ generation plants**

| **Transformation vector** | **Transgenic line** | **Plants of T0 generation** | | **Plants of T1 generation** | |
| --- | --- | --- | --- | --- | --- |
|  |  | **Type of target mutation** | **Phenotype** | **Phenotype of individual plants grown from non-fluorescent seeds** | **Phenotype of individual plants grown from fluorescent seeds** |
| FMPKC1-EUI1 | ZH11 (WT) | T1T1/T2T2/T3T3/T4T4 | Normal internode | Normal internode | - |
|  | eui1 1# | T1T1/t2t2/T3t3/t4T4 | Internode elongation | Not a single copy hemizygote, no planting | Not a single copy hemizygote, no planting |
|  | eui1 2# | t1t1/T2T2/T3t3/T4T4 | Internode elongation | All individual internode elongation | All individual internode elongation |
|  | eui1 3# | T1T1/t2T2/T3T3/t4t4 | Internode elongation | All individual internode elongation | All individual internode elongation |
|  | eui1 4# | T1T1/T2T2/T3t3/T4T4 | Normal internode | 28.13% individual internode elongation | 67.19% of individual internode elongation |
|  | eui1 5# | t1T1/T2T2/T3t3/T4T4 | Internode elongation | All individual internode elongation | All individual internode elongation |
| FMPKC2-PTC1 | YG13 (WT) | T1T1/T2T2 | Fertility | Fertility | - |
|  | ptc1 1# | T1t1/T2T2 | Fertility | 29.69% of the plants were sterile | 43.75% of the plants were sterile |
|  | ptc1 2# | T1t1/T2t2 | Fertility | 26.56% of the plants were sterile | 39.06% of the plants were sterile |
|  | ptc1 3# | t1t1/T2T2 | Sterility | No planting | No planting |
|  | ptc1 4# | T1T1/t2t2 | Sterility | No planting | No planting |
|  | ptc1 5# | T1T1/T2T2 | Fertility | 7.81% of the plants were sterile | 10.94% of the plants were sterile |
|  | ptc1 6# | T1T1/T2T2 | Fertility | No planting | No planting |
|  | ptc1 7# | t1T1/T2t2 | Sterility | No planting | No planting |
|  | ptc1 8# | T1T1/T2T2 | Fertility | No planting | No planting |
|  | ptc1 9# | T1T1/T2T2 | Fertility | No planting | No planting |

Note: T1, Target1; T2, Target2; T3, Target3; T4. Uppercase "T" means that the target sequence had no editing mutation, and lowercase "t" means that the target sequence had an editing mutation.

**Table S2 Copy number and proportion of non-fluorescent seeds of T**_0_ **positive lines**

| Samples | Number of molecules in 1ul DNA (*DsRed*) | Number of molecules in 1ul DNA (*OsSPS1*) | Molecular number ratio (*DsRed*/*OsSPS1*) | Proportion of non-fluorescent seeds | Single-copy hemizygote |
| --- | --- | --- | --- | --- | --- |
| eui1 #1 | 11880 | 7220 | 1.6454 | 11.76% | No |
| eui1 #2 | 5580 | 10760 | 0.5186 | 46.73% | Yes |
| eui1 #3 | 4080 | 7480 | 0.5455 | 45.97% | Yes |
| eui1 #4 | 16080 | 30840 | 0.5214 | 46.38% | Yes |
| eui1 #5 | 19680 | 39560 | 0.4975 | 45.64% | Yes |
| ptc1 #1 | 13960 | 27080 | 0.5155 | 50.61% | Yes |
| ptc1 #2 | 20320 | 39040 | 0.5205 | 50.00% | Yes |
| ptc1 #3 | 12680 | 26080 | 0.4862 | 49.38% | Yes |
| ptc1 #4 | 4660 | 8400 | 0.5548 | 51.32% | Yes |
| ptc1 #5 | 16260 | 31000 | 0.5245 | 50.66% | Yes |
| ptc1 #6 | 25840 | 55480 | 0.4658 | 50.59% | Yes |
| ptc1 #7 | 20960 | 39080 | 0.5363 | 48.81% | Yes |
| ptc1 #8 | 58800 | 116300 | 0.5056 | 49.17% | Yes |
| ptc1 #9 | 60300 | 128700 | 0.4685 | 48.74% | Yes |
| WT | 0 | 106300 | 0 | 100% | - |
| NTC | 0 | 0 | - | - | - |

**Note:** Three sterile lines, ptc1 3#, ptc1 4#, and ptc1 7#, were obtained by backcrossing with the WT, and the proportion of non-fluorescent seeds was subsequently analyzed. WT, wild-type; NTC, negative control.

**Table S3 Mutation types of T_0_ transgenic plants**

| **Vectors** | **Lines** | **DNA strands** | **Target1 sequence** | **Target2 sequence** | **Target3 sequence** | **Target4 sequence** |
| --- | --- | --- | --- | --- | --- | --- |
| FMPKC1-EUI1 | ZH11(WT) | Sense | 5'-GATCGCCGGGCTGTGCATTA-3' | 5'-CAAGTACCTCCAGAAAGGCC-3' | 5'-GGCTCCATCATCGAGAACAG-3' | 5'-GTTCCGGCTCATCATCGAGC-3' |
|  |  | Antisense | 3'-CTAGCGGCCCGACACGTAAT-5' | 3'-GTTCATGGAGGTCTTTCCGG-5' | 3'-CCGAGGTAGTAGCTCTTGTC-5' | 3'-CAAGGCCGAGTAGTAGCTCG-5' |
|  | eui1 #1 | Sense | 5'-GATCGCCGGGCTGTGCATTA-3' | 5'-CAAGTACCTCCAGAAAG**A**GCC-3' | 5'-GGCTCCATCATCGAGAACAG-3' | 5'-GTTCCGGCTCATCAT**--**AGC-3' |
|  |  | Antisense | 3'-CTAGCGGCCCGACACGTAAT-5' | 3'-GTTCATGGAGGTCTTTC**T**CGG-5' | 3'-CCGAGGTAGTAGCTCTT**G**GTC-5' | 3'-CAAGGCCGAGTAGTAGCTCG-5' |
|  | eui1 #2 | Sense | 5'-GATCGCCGGGCTGTGC**-**TTA-3' | 5'-CAAGTACCTCCAGAAAGGCC-3' | 5'-GGCTCCATCATCGAGAACAG-3' | 5'-GTTCCGGCTCATCATCGAGC-3' |
|  |  | Antisense | 3'-CTAGCGGCCCGACACGTAAT-5' | 3'-GTTCATGGAGGTCTTTCCGG-5' | 3'-CCGAGGTAGTAGCTCTT**G**GTC-5' | 3'-CAAGGCCGAGTAGTAGCTCG-5' |
|  | eui1 #3 | Sense | 5'-GATCGCCGGGCTGTGCATTA-3' | 5'-CAAGTACCTCCAGAAAG**A**GCC-3' | 5'-GGCTCCATCATCGAGAACAG-3' | 5'-GTTCCGGCTCATCATCGAGC-3' |
|  |  | Antisense | 3'-CTAGCGGCCCGACACGTAAT-5' | 3'-GTTCATGGAGGTCTTTCCGG-5' | 3'-CCGAGGTAGTAGCTCTTGTC-5' | 3'-CAAGGCCGAGTAGTAGC**C**TCG-5' |
|  | eui1 #4 | Sense | 5'-GATCGCCGGGCTGTGCATTA-3' | 5'-CAAGTACCTCCAGAAAGGCC-3' | 5'-GGCTCCATCATCGAGAACAG-3' | 5'-GTTCCGGCTCATCATCGAGC-3' |
|  |  | Antisense | 3'-CTAGCGGCCCGACACGTAAT-5' | 3'-GTTCATGGAGGTCTTTCCGG-5' | 3'-CCGAGGTAGTAGCTCT**-**GTC-5' | 3'-CAAGGCCGAGTAGTAGCTCG-5' |
|  | eui1 #5 | Sense | 5'-GATCGCCGGGCTGTGCA**TC**TTA-3' | 5'-CAAGTACCTCCAGAAAGGCC-3' | 5'-GGCTCCATCATCGAGAACAG-3' | 5'-GTTCCGGCTCATCATCGAGC-3' |
|  |  | Antisense | 3'-CTAGCGGCCCGACACGT**AG**AAT-5' | 3'-GTTCATGGAGGTCTTTCCGG-5' | 3'-CCGAGGTAGTAGCTCTT**A**GTC-5' | 3'-CAAGGCCGAGTAGTAGCTCG-5' |
| FMPKC2-PTC1 | YG13 (WT) | Sense | 5'-CATGGTGGTCACCAAGTACC-3' | 5'-CGAGCTGCGCGCCCGCCTGC-3' |  |  |
|  |  | Antisense | 3'-GTACCACCAGTGGTTCATGG-5' | 3'-GCTCGACGCGCGGGCGGACG-5' |  |  |
|  | ptc1 #1 | Sense | 5'-CATGGTGGTCACCAAGTACC-3' | 5'-CGAGCTGCGCGCCCGCCTGC-3' |  |  |
|  |  | Antisense | 3'-GTACCACCAGTGGTTCA**A**TGG-5' | 3'-GCTCGACGCGCGGGCGGACG-5' |  |  |
|  | ptc1 #2 | Sense | 5'-CATGGTGGTCACCAAGTACC-3' | 5'-CGAGCTGCGCGCCCGCCTGC-3' |  |  |
|  |  | Antisense | 3'-GTACCACCAGTGGTT**--**TGG-5' | 3'-GCTCGACGCGCGGGCGG**A**ACG-5' |  |  |
|  | ptc1 #3 | Sense | 5'-CATGGTGGTCACCAAGT**T**ACC-3' | 5'-CGAGCTGCGCGCCCGCCTGC-3' |  |  |
|  |  | Antisense | 3'-GTACCACCAGTGGTTCA**A**TGG-5' | 3'-GCTCGACGCGCGGGCGGACG-5' |  |  |
|  | ptc1 #4 | Sense | 5'-CATGGTGGTCACCAAGTACC-3' | 5'-CGAGCTGCGCGCCCGCCTGC-3' |  |  |
|  |  | Antisense | 3'-GTACCACCAGTGGTTCATGG-5' | 3'-GCTCGACGCGCGGGCGGACG-5' |  |  |
|  | ptc1 #5 | Sense | 5'-CATGGTGGTCACCAAGTACC-3' | 5'-CGAGCTGCGCGCCCGCCTGC-3' |  |  |
|  |  | Antisense | 3'-GTACCACCAGTGGTTCATGG-5' | 3'-GCTCGACGCGCGGGCGGACG-5' |  |  |
|  | ptc1 #6 | Sense | 5'-CATGGTGGTCACCAAGTACC-3' | 5'-CGAGCTGCGCGCCCGCCTGC-3' |  |  |
|  |  | Antisense | 3'-GTACCACCAGTGGTTCATGG-5' | 3'-GCTCGACGCGCGGGCGGACG-5' |  |  |
|  | ptc1 #7 | Sense | 5'-CATGGTGGTCACCAA**--**ACC-3' | 5'-CGAGCTGCGCGCCCGCCTGC-3' |  |  |
|  |  | Antisense | 3'-GTACCACCAGTGGTTCATGG-5' | 3'-GCTCGACGCGCGGGCGG**C**ACG-5' |  |  |
|  | ptc1 #8 | Sense | 5'-CATGGTGGTCACCAAGTACC-3' | 5'-CGAGCTGCGCGCCCGCCTGC-3' |  |  |
|  |  | Antisense | 3'-GTACCACCAGTGGTTCATGG-5' | 3'-GCTCGACGCGCGGGCGGACG-5' |  |  |
|  | ptc1 #9 | Sense | 5'-CATGGTGGTCACCAAGTACC-3' | 5'-CGAGCTGCGCGCCCGCCTGC-3' |  |  |
|  |  | Antisense | 3'-GTACCACCAGTGGTTCATGG-5' | 3'-GCTCGACGCGCGGGCGGACG-5' |  |  |

WT, wild-type

**Table S4 Primer characteristics**

| **Primer name** | **Forward (5'-3')** | **Reverse (5'-3')** |
| --- | --- | --- |
| **Construction of FMPKC expression vectors and gRECs cloning vectors** | | |
| Adapter-EA | cgcgccgataccgcgagacccacgctcacttaatacagggcgcgtccattcgccacttcagaggtctctcgagcactcgtggatggacaatttaaattccgttttacct | cgcgaggtaaaacggaatttaaattgtccatccacgagtgctcgagagacctctgaagtggcgaatggacgcgccctgtattaagtgagcgtgggtctcgcggtatcgg |
| *DsRed2* | AGCACCGGTAAAAGGCGCGAAACCGTCTCTTCGTGAGAATAACCGTGGCCTAAAAATAAGCC | GTCTCGCGGTATCGGCGCGCCGGCCGCATTCGCAAAACACACCTAGACTA |
| *Pollen killer* | TGCGAATGCGGCCGGCGCGACAGGCATACCAGACAGTCCGGTGTGC | GTCTCGCGGTATCGGCGCGGGAGATATAGGGGAAAGAGAACGCTGATGTGACAAGTGAG |
| gRNA | CCAACTGTCGACGTTTTAGAGCTAGAAATAGCAAGTTAAAATAAGGCTAG | TGAACTAAGCTTAAAATTCCATCCACTCCAAGCTCTTG |
| 1.8kb spacing | CTCTGTGGTACCAATCGGCATCTGAATATCCTATTAAATC | CCAACTGTCGACGTCCCGCCCTCGAAGATGCAGTAC |
| U3 Promoter | TGAACTGAATTCTCCGTTTTACCTGTGGAATCGGCAGC | CTCTGTGGTACCTGCCACGGATCATCTGCACAACTCT |
| U6a Promoter | TGAACTGAATTCTCCGTTTTACCTGTGGAATCGGCAGC | CTCTGTGGTACCCGGCAGCCAAGCCAGCACC |
| U6b Promoter | TGAACTGAATTCTCCGTTTTACCTGTGGAATCGGCAGC | CTCTGTGGTACCCAACACAAGCGGCAGCGCG |
| U6c Promoter | TGAACTGAATTCTCCGTTTTACCTGTGGAATCGGCAGC | CTCTGTGGTACCCTGAGCCTCAGCGCAGCAGC |
| **Construction of FMPKC1-EUI1 and FMPKC2-PTC1 gene editing vectors** | | |
| U3ET1-Adapter | AGATGATCCGTGGCAGATCGCCGGGCTGTGCATTAGTTTTAGAGCTAGAA | TTCTAGCTCTAAAACTAATGCACAGCCCGGCGATCTGCCACGGATCATCT |
| UaET2-Adapter | CTGGCTTGGCTGCCGCAAGTACCTCCAGAAAGGCCGTTTTAGAGCTAGAA | TTCTAGCTCTAAAACGGCCTTTCTGGAGGTACTTGCGGCAGCCAAGCCAG |
| UbET3-Adapter | CTGCCGCTTGTGTTGGGCTCCATCATCGAGAACAGGTTTTAGAGCTAGAA | TTCTAGCTCTAAAACCTGTTCTCGATGATGGAGCCCAACACAAGCGGCAG |
| UcET4-Adapter | TGCGCTGAGGCTCAGGTTCCGGCTCATCATCGAGCGTTTTAGAGCTAGAA | TTCTAGCTCTAAAACGCTCGATGATGAGCCGGAACCTGAGCCTCAGCGCA |
| U3PT1-Adapter | AGATGATCCGTGGCACATGGTGGTCACCAAGTACCGTTTTAGAGCTAGAA | TTCTAGCTCTAAAACGGTACTTGGTGACCACCATGTGCCACGGATCATCT |
| UaPT2-Adapter | CTGGCTTGGCTGCCGCGAGCTGCGCGCCCGCCTGCGTTTTAGAGCTAGAA | TTCTAGCTCTAAAACGCAGGCGGGCGCGCAGCTCGCGGCAGCCAAGCCAG |
| CDA | CGTGGATGGACAATTTAAATTCCGTTTTACCTCTCGTAGTCCAGGTGTGACTCGCAC | CAGGTAAAACGGAATTTACGACCATCCACTCCAAGCTCTTG |
| GGL1 | TTCAGAGGTCTCTCTCGCTCGTAGTCCAGGTGTGACTCGCAC | AGCGTGGGTCTCGTCAGACGACCATCCACTCCAAGCTCTTG |
| GGL2 | TTCAGAGGTCTCTCTGACTCGTAGTCCAGGTGTGACTCGCAC | AGCGTGGGTCTCGACCGACGACCATCCACTCCAAGCTCTTG |
| FPC-J | GTTCCCCCTAAATTTCTCCCCCTATATCTCACTCACTTGTCACATCAG | CTCCGCACCCGACATAGATGCAATAACTTCGTATAGGCTAATTTATACG |
| **Validation of positive transgenic plants** | | |
| DsRed2-J | GAATGGAGTCGTCTGCTTGCTAGCCTTCGCCTAC | GAAGTTCACGCCGATGAACTTCACCTTGTAGATGAA |
| EUI1-T1-S | AGAAGGGCGAGGTGAGCGAGGCGAGAC | CCTCCACTTGTGGAAGTAAGGGAAGATCCTGGTGCA |
| EUI1-T2-S | TGACGTTTAACTTGATTCATCATTCGGCGACGTCTCTTG | TCACTCCCAAAGCAAGCCCGCGATATCACATCGAAG |
| EUI1-T3-S | GAGACCAGCGTCATCTTCAGCATCCCTTCGCT | CGCTCGTCTCGTGCCCGGCGAAGTAG |
| EUI1-T4-S | ACTTCGACATGGTGTCCCGGATGCGGAC | GAACTATGCACGGGTGCAGTAGATGTGTCAACTTG |
| PTC1-T1-S | ACCAGATCATGGACCTCTGGGACCGCATTTG | AGGAGGTCGCCGAGGCTGAGCAGCTTGTG |
| PTC1-T2-S | AGCGGCCACAAGCTGCTCAGCCTC | CTTTGACGAAGTGCTTGATGTCCAGGACCATCCG |
| **Estimation of T-DNA copy number by ddPCR** | | |
| DsRed-ddPCR | CCCAGTTCCAGTACGGCTC | ACGCGCTCCCACTTGAA |
| DsRed-P | 5’FAM-ACATCCCCGACTACA-3’MGB | |
| SPS1-ddPCR | ACTCGGTGCAGAGGATCATG | GCGGAGGAGCGGGTACT |
| SPS1-P | 5’FAM-ATCAAGCGGTCGTCT-3’MGB | |
| **Detection of genetically modified components in non-fluorescent seeds** | | |
| HPT-K | FAM-GAAGGTGACCAAGTTCATGCTCCGCAAGGAATCGGTCAAT | VIC-GAAGGTCGGAGTCAACGGATTGATCAGCAATCGCGCATATG |
| Cas9-K | FAM-GAAGGTGACCAAGTTCATGCTACTCTCTTCGAGGATAG | VIC-GAAGGTCGGAGTCAACGGATTGCCTACCCCAACCGGTGTAA |
| PLD-K | FAM-GAAGGTGACCAAGTTCATGCTCAGCTCATTTGCATGGAAAGCCGAT | VIC-GAAGGTCGGAGTCAACGGATTGACTGCAAAACGCTCACCAGCTTC |

**Supplemental Materials and Methods**

**1. Construction of FMPKC expression vectors and gRECs cloning vectors**

The fluorescence marker and pollen killer-assisted CRISPR/Cas9 (FMPKC) expression vectors mainly contained linked *Cas9*, *DsRed2*, *RAmy1A,* or *orfH79* expression cassettes and two assembly entrances for gRECs (gRNA expression cassettes). Detailed information on the *Cas9*, *DsRed2,* and *orfH79* expression cassettes can be found in previous studies (Ma et al., 2015; Song et al., 2021). The modified *RAmy1A* (Ouyang et al., 2021), whose signal peptide was replaced with an amyloplast-targeting transit peptide from the maize *Brittle-1* gene, was driven by the pollen-specific promoter Pg47 to form a novel transgenic pollen killer (Figure S8). DsRed2 is controlled by the endosperm-specific promoter Ltp (barley lipid transport protein gene promoter). It was predominantly expressed in the endosperm but not in the husk. The Golden Gate cloning entrance and the Cyclic Digestion and Assembly entrance were realized by introducing two BsaI sites and a SwaI site, respectively.

FMPKC plasmids were constructed on the pYLCRISPR/Cas9-MH vector (pMH), which has been widely used for gene editing in monocot plants (Ma et al., 2015). All the assembly entrances and expression cassettes were assembled at the AscI site of the pMH vector using a seamless cloning method (Yu et al., 2017). First, we removed the original BsaI sites of pMH by AscI digestion and ligation of adapter DNA containing the Golden Gate cloning entrance and Cyclic Digestion and Assembly entrance to form the intermediate vector pMH-AE. The *DsRed2* expression unit was then recombined into the reserved AscI site of the pMH-AE vector, yielding the plasmid, pMH-DsRed2. Finally, *RAmy1A* and *orfH79* transgene pollen killers were assembled into the reserved AscI site of the pMH-DsRed2 plasmid, resulting in plasmids FMPKC1 and FMPKC2, respectively.

Four gRECs cloning vectors were constructed based on the pUC57-Kan plasmid. We first amplified the gRNA scaffold (containing 7bp PolyT) and ligated it to the pUC57-Kan vector digested with SalI and HindIII, forming the intermediate vector pK-gRNA. Second, a 1.8-kb spacing sequence was cloned into pK-gRNA digested with KpnI and SalI, yielding the plasmid pK-Spacing-gRNA. Finally, four rice snRNA promoters (U3/U6a/U6b/U6C) were digested with EcoRI and KpnI and ligated to pK-Spacing-gRNA to obtain four gRECs cloning vectors, pCR-U3/U6a/U6b/U6C, which were used for assembly of the target site sequence.

The primers used here are listed in Table S4.

**2. Construction of FMPKC1-EUI1 and FMPKC2-PTC1** **gene editing vectors**

The CRISPR-GE web tool, available at http://skl.scau.edu.cn, was used to design four targets on exons for EUI1 and two targets on exons for PTC1. It should be noted that the above targets need to be designed for a specific location; therefore, they are not the highest-rated targets in the CRISPR-GE software. For instance, when targeting EUI1, the distances between the four targets of EUI1 must be considered to facilitate PCR amplification and sequencing. When targeting PTC1, it is necessary to design two targets near the original mutation site to avoid unexpected thermosensitive sterility traits. These targets were designated as EUI1-Target1, EUI1-Target2, EUI1-Target3, EUI1-Target4, PTC1-Target1, and PTC1-Target2, and Figure S3a shows their positions on the exon. Based on the promoter of the gRECs cloning vector (Figure S2b), the corresponding 15 bp overlapping sequence was added on both sides of the target to form the target site adapter primers (Table S4). Target site adapter primers were synthesized, diluted, and mixed in equal concentrations with the forward and reverse primers. The mixture was incubated at 95 ℃ for 3 min and annealed to 30 ℃ to form a target site adapter with a final concentration of 10 uM. Based on the overlapping sequences at the target site, appropriate primers, gRNA-F and U3-R (U6a-R/U6b-R/U6c-R), were selected to amplify the corresponding gRECs cloning vector, and linearized gRECs vectors were generated. Gibson Assembly was performed to ligate the linearized gRECs vector and target site adapter to form a complete gRECs clone plasmid containing the target site sequence. After the transformation of *Escherichia coli*, PCR detection of the plasmids was performed using M13 primers, and single colonies with amplification bands of 618 bp (U3)/683 bp (U6a)/569 bp (U6b)/978 bp (U6a) were screened. These colonies contained complete gRECs cloning plasmids containing the target sites (pCR-U3-EUI1-T1, pCR-Ua-EUI1-T2, pCR-Ub-EUI1-T3, pCR-Uc-EUI1-T4, pCR-U3-PTC1-T1, and pCR-Ua-PTC1-T2).

Four gRECs of *EUI1* were recombined into SwaI sites of the FMPKC1 vector using the Cyclic Digestion and Assembly method. The procedure was as follows: (1) Four complete gRECs cloned vectors containing *EUI1* target site were used as templates for PCR amplification using CDA-F and CDA-R primers to obtain linearized gRNA expression cassettes (U3-EUI1-T1-gRNA, U6a- EUI1-T2-gRNA, U6b-EUI1-T3-gRNA, and U6c-EUI1-T4-gRNA). (2) SwaI digestion of FMPKC1 vector. (3) Gibson Assembly of the linearized U3-EUI1-T1-gRNA and FMPKC1 digestion products was performed to obtain the intermediate plasmid FMPKC1-EUI1-T1. (4) Because the primer CDA-F contains the Cyclic Digestion Assembly entrance sequence, when the amplified gRECs were assembled into FMPKC1, a SwaI enzyme site was regenerated upstream of the first gRECs of the intermediate plasmid, which could be used for the assembly of the second gRECs. (5) Similarly, when the second gREC is assembled into the intermediate plasmid, Cyclic Digestion and Assembly entrance are regenerated to assemble the next gRECs. Therefore, the four EUI1 gRECs were sequentially inserted into FMPKC1 through a cyclic operation involving SwaI digestion and Gibson Assembly. The resulting vector was named FMPKC1-EUI1-T1-T2-T3-T4. For simplicity, this vector was referred to as FMPKC1-EUI. To determine the maximum quantity of gRECs that could be loaded into FMPKC, we conducted a new Cyclic Digestion and Assembly round using four gRECs. During the second round, only gRECs U3-EUI1-T1-gRNA, Ua-EUI1-T2-gRNA, and Ub-EUI1-T3-gRNA were successfully assembled into FMPKC1-EUI1. Consequently, we obtained a seven-target vector: FMPKC1-EUI-T1-T2-T3-T4-T1-T2-T3. Figure S1 shows a Schematic diagram of the Cyclic Digestion and Assembly.

Two gRECs of the *PTC1* gene were ligated into FMPKC2 using the Golden Gate cloning method. Primers GGL1-F and GGL1-R were used to amplify the linearized gREC of U3-PTC1-T1-gRNA, and primers GGL2-F and GGL2-R were used to amplify the linearized gREC of U6a-PTC1-T2-gRNA. Following the procedure described by Ma et al. (2015), the linearized gRECs of U3-PTC1-T1-gRNA and U6a-PTC1-T2-gRNA were ligated with FMPKC2 in one step to construct the final expression vector, FMPKC2-PTC1.

**3. Plant transformation and growth**

The FMPKC1-EUI1 and FMPKC1-PTC1 plasmids were transformed into ZH11 (Zhonghua11) and YG13 (Yangeng13) (*O. sativa* L. ssp. japonica), respectively, through *A. tumefaciens* -mediated transformation (Hiei et al., 1994). Resistant calluses were screened using a 30 mg/L Hygromycin B solution. All rice plants were grown in a greenhouse under a photoperiod of 16 h light/8 h dark.

**4. Validation of positive transgenic plants**

Genomic DNA was extracted from the rice plants using the CTAB method. Positive transgenic lines in the T_0_ generation were identified during the seedling stage by amplifying the DsRed2 gene expression cassettes using DsRed-J-F and DsRed-J-R primers. Furthermore, fluorescence was observed in mature seeds to validate the PCR results of the positive transgenic lines. Sanger sequencing was conducted on the transgenic plants to analyze the mutation types. Initially, mutation types were checked using the DSDecodeM tool, a web-based application that efficiently decodes multiple overlapping sequencing chromatograms (Xie et al., 2017). Next, TA cloning and sequencing analyses were performed to confirm the genome-edited sites. The primers used for amplification and sequencing of the target sites are listed in Table S4. The targets were considered to be edited once target sequences were changed without considering mutation type and are represented by lowercase “t” Unchanged target sites are indicated by uppercase “T” (Table S1).

**5. Estimation of T-DNA copy number in T**_0_**-positive transgenic plants**

The absolute number of copies of exogenous gene *DsRed2* and rice endogenous single copy gene *OsSPS1* (Hirose et al., 2014) in each sample was detected through Droplet Digital PCR (ddPCR, QX200™ Droplet Digital™ PCR platform, Bio-Rad) as described previously (Hindson et al., 2021). The T-DNA copy numbers and insertion states (homozygous or hemizygous) of T_0_-positive transgenic plants were determined using the copy ratio of *DsRed2* and *OsSPS1*. Table S4 lists the Droplet Digital PCR primers and probes for *DsRed2* and *OsSPS1*.

**6. Observation of inactive pollen and fluorescent seeds**

One day before flowering, the anthers of the wild-type and fertile single-copy hemizygotic transgenic lines were collected. After staining with 1% KI-I, pollen was released from the broken anthers. The round brownish-black pollen grains indicate active pollen. Irregular and transparent pollen grains indicated the presence of inactive pollen. The proportion of inactive light-stained pollen in the wild-type and transgenic lines was counted under a microscope. The counting was repeated thrice for each plant.

The fluorescent seeds of the FMPKC1-EUI1 and FMPKC2-PTC1 fertile lines were directly observed and sorted using a LUYOR-3415RG portable fluorescent protein excitation light source and LUV-50A viewing glasses. Fluorescent imaging was conducted by placing the corresponding red filter in front of the camera lens. The FMPKC2-PTC1 sterile line was backcrossed with wild-type YG13, and the ratio of fluorescent seeds was calculated.

**7. Analysis of the lethality rate of pollen killer**

The lethality rates of pollen killers (RAmyA1/orfH79) were calculated in two ways according to the ratio of inactive and non-fluorescent seeds.

a) Calculated using the ratio of inactive pollen. Single-copy hemizygote transgenic lines can produce 50% transgenic pollen according to Mendel's genetic law, and transgenic pollens containing pollen killers are inactive: therefore, there are inactive and active pollens in transgenic plants. We assumed that the pollen killer lethality rate was R, the ratio of transgenic inactive pollen was P_1_^i^ and the ratio of transgenic active pollen was P_1_^a^. Theoretically, we can conclude that P_1_^i^ + P_1_^a^ = 50%, and R = P_1_^i^/(P_1_^i^ + P_1_^a^) = P_1_^i^/50% = 2P_1_^i^. Under natural conditions, few pollen grains of wild-type plants might be inactivated, which might be caused by the genetic background and environment; the ratio of inactivated pollen in wild-type plants was designated as P_0_^i^. The ratio of transgenic inactive pollen caused by pollen killers was P_1_^i^ = P^i^ - P_0_^i^, so the final formula was R = (P^i^-P_0_^i^)/50% = 2(P^i^-P_0_^i^). P_0_^i^ and P^i^ are the ratios of inactive pollen in the wild-type and transgenic plants, respectively.

b) Calculated using the ratio of non-fluorescent seeds. The pollen killer lethality rate was defined as R. Single-copy hemizygote transgenic lines, whose genotype was A-, could generate progeny with three genotypes (AA, A-, and--), with proportions of 25%, [25% + 25% (1-R)], and 25%, respectively (Table 1). It is assumed that the ratio of non-fluorescent seeds in transgenic lines is P^n^; theoretically, P^n^ = 25%/[50% + 50%(1-R)]. Finally, we calculated the real lethality rate according to the formula R = 2-1/(2P^n^).

**Table S5 Genotypes and proportion of segregated progeny of single copy hemizygotes with pollen killer**

| MG  FM | 50% (1-R) A | 50% - |
| --- | --- | --- |
| 50% A | 25% (1-R) AA | 25% A- |
| 50% - | 25% (1-R) A- | 25% -- |

**Note:** MG, male gametophytes; FG, female gametophytes; R, lethality rate of pollen killer; A, the genotype of gametophytes with exogenously linked expression elements of *Cas9^DsRed^*^-^*^Pollen killer^*; - indicates the genotype of gametophytes without *Cas9^DsRed^*^-^*^Pollen killer^*.

**8. Detection of genetically modified components in non-fluorescent seeds**

The T-DNA of the FMPKC vector contained closely linked *HPT*, *Cas9*, *DsRed2,* and pollen-killer expression elements. This design enables the screening of non-fluorescent seeds for those containing exogenous transgenic components by detecting one or two expression elements. *HPT* and *Cas9* were detected using the KASP technology. The housekeeping gene *PLD* was used as a reference gene. Table S4 shows the sequence of markers and the 5' terminal modification. The instruments, reagents, and reaction procedures used were based on the methods of He et al. (2014), 465–510 and 618–660 nm indicates the fluorescence detection signals of exogenous genes (*HPT* and *Cas9*) and the housekeeping gene *PLD*, respectively.

**9. Statistical analysis**

GraphPad Prism 8.0 and Excel 2019 were used for the analysis of variance, significant analysis, and quantification of the data. Student’s test and Turkey’s multiple comparison test were used to detect any significant difference (* P <0.05, ** P <0.01, and *** P <0.001). Data are presented as mean ± standard deviations according to data from experiments repeated at least three times.

**References for Supplemental Materials and Methods**

He CL, Holme J, Anthony J (2014) SNP genotyping: the KASP assay. *Methods in Molecular Biology* **1145**: 75–86.

Hiei Y, Ohta S, Komari T, et al. (1994) Efficient transformation of rice (*Oryza sativa* L.) mediated by Agrobacterium and sequence analysis of the boundaries of the T-DNA. *The Plant journal* **6**: 271–282.

Hindson BJ, Ness KD, Masquelier DA, et al. (2021) Pyramiding favorable alleles in an elite wheat variety in one generation by CRISPR-Cas9-mediated multiplex gene editing. *Molecular plant* **14**: 847–850.

Hirose T, Hashida Y, Aoki N, et al. (2014) Analysis of gene-disruption mutants of a sucrose phosphate synthase gene in rice, *OsSPS1*, shows the importance of sucrose synthesis in pollen germination. *Plant science* **225**: 102–106.

Ma XJ, Zhang QY, Zhu QL, et al. (2015) A robust CRISPR/Cas9 system for convenient, high-efficiency multiplex genome editing in monocot and dicot plants. *Molecular plant* **8**: 1274–1284.

Ouyang N, Sun XW, Tan YN, et al. (2021) Senescence-specific expression of RAmy1A accelerates non-structural carbohydrate remobilization and grain filling in rice (*Oryza sativa* L.). *Frontiers in Plant Science* **12**: 647574.

Song SF, Wang TK, Li YX, et al. (2021) A novel strategy for creating a new system of third-generation hybrid rice technology using a cytoplasmic sterility gene and a genic male-sterile gene. *Plant Biotechnology Journal* **19**: 251–260.

Xie X, Ma X, Zhu Q, et al. (2017) CRISPR-GE: A Convenient Software Toolkit for CRISPR-Based Genome Editing. *Molecular Plant* **10**: 1246-1249.

Yu D, Tan YN, Sun ZZ, et al. (2017) In vitro seamless stack enzymatic assembly of DNA molecules based on a strategy involving splicing of restriction sites. *Scientific reports* **7**: 14261.
